# Feasibility and acceptability of the World Health Organization’s iSUPPORT program for dementia caregivers in Uganda

**DOI:** 10.20935/mhealthwellb7292

**Published:** 2024-08-12

**Authors:** Joy Louise Gumikiriza-Onoria, Roy William Mayega, Janet Nakigudde, Bruno Giordani, Martha Sajatovic, Mark Kaddu Mukasa, Dennis Buwembo, Kamada Lwere, Noeline Nakasujja

**Affiliations:** 1 Department of Psychiatry, School of Medicine, Makerere University College of Health Sciences, Kampala 7072, Uganda.; 2 School of Public Health, Makerere University College of Health Sciences, Kampala 7072, Uganda.; 3 Department of Psychiatry, University of Michigan Faculty Ombuds, Ann Arbor, MI 48104, USA.; 4 School of Medicine, Case Western Reserve University, Cleveland, OH 44106, USA.; 5 School of Medicine, Makerere University College of Health Sciences, Kampala 7072, Uganda.; 6 School of Biomedical Sciences, Makerere University College of Health Sciences, Kampala 7072, Uganda.

**Keywords:** Alzheimer’s disease and related dementias (ADRD), low- and middle-income countries (LMICs), caregiver support, psychological distress, quality of life (QoL), feasibility and acceptability

## Abstract

This study aims to address Alzheimer’s disease and related dementias (ADRD) in low- and middle-income countries (LMICs). It involves adaptation of the World Health Organization’s iSUPPORT (WHO-iSUPPORT) psychosocial intervention for Uganda, assessing its feasibility and acceptability, and its effects on caregivers’ psychological distress, quality of life (QoL), and depression levels. The adapted iSUPPORT (UGA-iSUPPORT) program was translated into Luganda, a local language, validated by experts, divided into four modules, and later piloted for four weeks in Wakiso, Uganda. The caregivers were randomly assigned to receive weekly UGA-iSUPPORT sessions or modified standard care via weekly phone consultations. Baseline and endpoint changes were assessed using the Kessler Psychological Distress Scale (K-10), the Centre for Epidemiological Studies Depression Scale (CES-D), and the Measure of Quality of Life for Dementia Caregivers (C-DEMQOL). The intervention group (*n* = 33, 87.9% females, mean age 32.5 years) showed significant improvements in psychological distress (decreased from 29.2 to 23.7, *p* = 0.001), depression (from 33.4 to 25.6, *p* = 0.001), and QoL (from 81.1 to 89.4, *p* = 0.001). The control group (*n* = 32, 50% female, mean age 36.7 years) did not experience similar enhancements. The high retention rate (97%) and positive feedback from the participants underscored the program’s feasibility and acceptability. UGA-iSUPPORT effectively boosted the mental health and well-being of dementia caregivers in LMICs. This study highlights the necessity of enduring and expandable interventions within healthcare systems. Further studies are warranted to examine these interventions’ prolonged impacts.

## 1. Introduction

Dementia is a major public health issue and a social and economic challenge in low- and middle-income countries (LMICs), where the number of people living with the condition is projected to increase rapidly in the coming years [1]. The number of people with dementia in Uganda, a sub-Saharan African country, is expected to increase from 98,000 in 2015 to 131,000 by 2025 [1, 2]. However, LMICs, such as Uganda, face several barriers to providing adequate care and support for people with dementia and their caregivers, such as limited healthcare resources, poor dementia awareness, and the cultural stigma associated with mental health [3–6].

Informal caregivers of people with dementia in LMICs experience high levels of psychological distress, including stress, depression, and burnout, due to the emotional and physical demands of caregiving as well as the lack of access to professional and social support [5]. These factors negatively affect the quality of life (QoL) and well-being of both caregivers and care recipients and pose a threat to the sustainability of informal care systems in countries such as Uganda. Therefore, there is an urgent need for culturally sensitive and resource-appropriate interventions to enhance the skills, knowledge, and coping strategies of caregivers of patients with dementia in Uganda.

The World Health Organization’s iSUPPORT (WHO-iSUPPORT) program is a comprehensive training and support initiative for dementia caregivers, based on cognitive-behavioral and problem-solving approaches [7, 8]. The program is effective in improving caregiver outcomes in various settings such as Australia, India, China, and the United Kingdom [9–14]. However, the program requires adapting to the local context and needs of the target population to ensure its acceptability, feasibility, and fidelity. Patel et al. [15] highlighted the crucial role of culturally tailored interventions in global mental health, particularly in caregiver support. For example, adapting interventions to include local idioms of distress, culturally relevant coping strategies, and community-based delivery modes can improve the acceptability and effectiveness of interventions for caregivers of patients with chronic conditions, such as dementia. These adaptations extend their impact beyond individual caregivers and contribute to a more supportive environment for mental health within the community.

In response to this need, the iSUPPORT program was adapted in this study for the Ugandan context, involving simplification of language and the incorporation of locally relevant examples. This adaptation aimed to enhance the accessibility and suitability of the program for Ugandan caregivers. This localized version of iSUPPORT, referred to as the adapted iSUPPORT (UGA-iSUPPORT), contributes to the growing body of evidence supporting the global utility and scalability of the iSUPPORT program and serves as a model for future adaptations in similar settings.

This study further aims to evaluate the UGA-iSUPPORT program to assess its feasibility, fidelity, acceptability, and impact on caregivers’ psychological distress; QoL; and depression severity for caregivers of people with dementia in Uganda. The results of this study provide vital insights into future interventions for dementia caregivers in Uganda and other LMICs.

## 2. Materials and methods

### Study design:

This was a four-week pilot randomized, unblinded, parallel intervention-control group trial (RCT) assessing the effectiveness of the UGA-iSUPPORT program on mental well-being in comparison with modified standard care among family caregivers of patients with Alzheimer’s disease and related dementias (ADRD) in the Wakiso District of Uganda. It focuses on key outcomes, such as caregiver distress, depression, and QoL, among family caregivers of patients with ADRD. The study cohort was drawn from an existing group initially established for the community assessment of ADRD, along with caregivers of individuals with confirmed ADRD diagnoses through detailed evaluations by psychiatrists and psychologists. The study focuses on caregivers who had been living with patients with ADRD in the same household or nearby for at least the past 12 months, were aged 18 years or older, and were not too ill to limit their participation in the study. Using random sampling techniques, 65 caregivers were selected and further randomized into either the intervention or control group using simple random selection. The intervention group engaged in four weekly sessions of the UGA-iSUPPORT program, which were specifically tailored to address the emotional and practical needs of caregivers. Meanwhile, the control group received four weekly phone consultations providing empathy and asking about patient well-being. To minimize the interaction and contamination between caregivers in the intervention and control groups, participants were selected from different sub-counties and villages to ensure geographical separation. Intervention sessions were scheduled at times that were distinct from the control group activities to prevent incidental meetings. The study team closely monitored attendance and interactions, reinforced confidentiality, and established separate communication channels for each group. This study measured feasibility, fidelity, and acceptability through comprehensive recruitment data, participant engagement, fidelity checklists, and direct observations, thus providing a detailed account of the program’s implementation [16, 17]. To evaluate the UGA-iSUPPORT’s effectiveness and its impact on caregivers’ well-being, baseline and end line data were collected assessing levels of psychological distress, symptoms suggestive of depression, and evaluation of QoL.

### Selection criteria:

**Figure 1 •:**
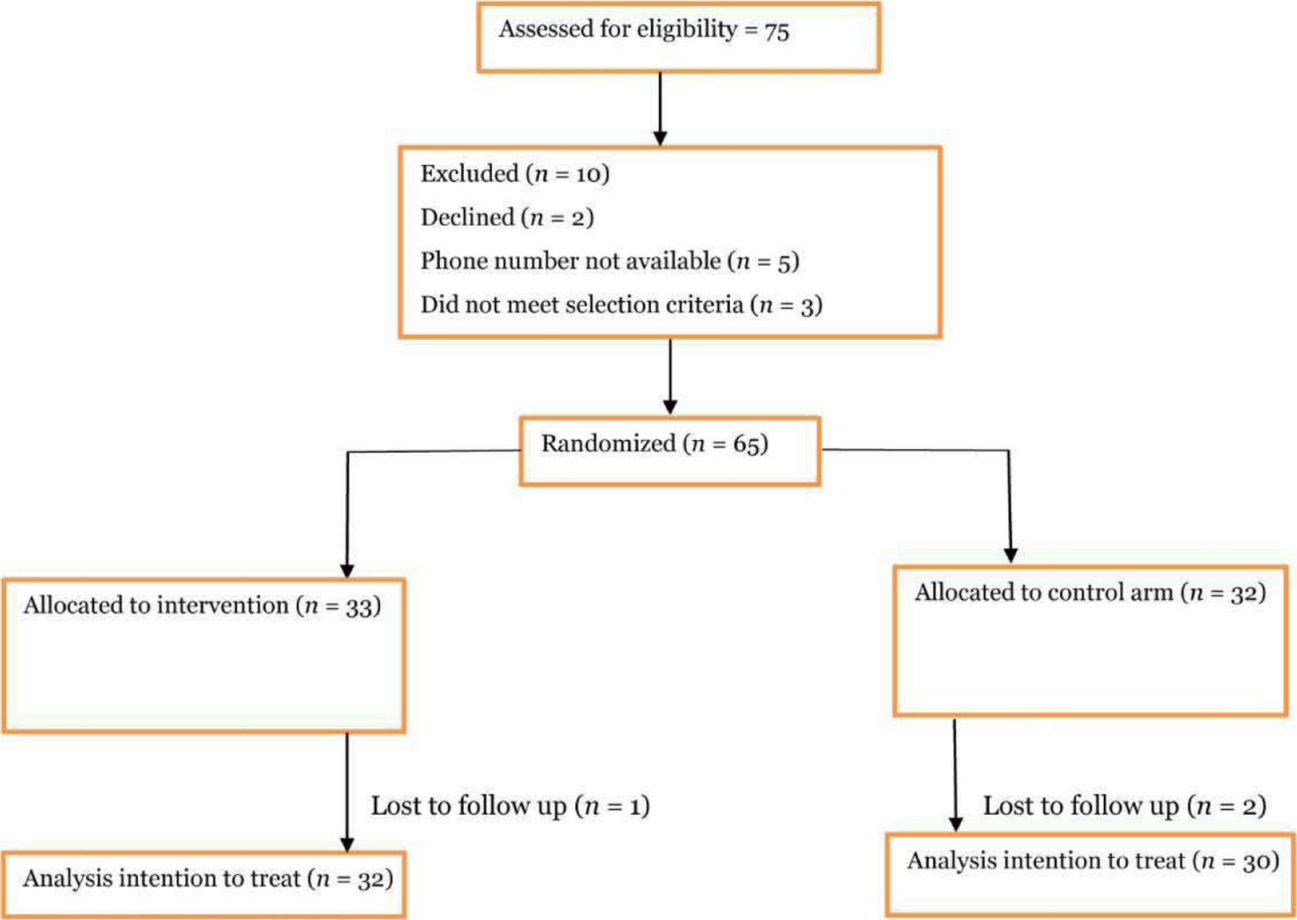
Participants’ flow chart.

In this pilot study, we utilized a modified simple random sampling technique, following Hinkelmann et al. [18], to select 65 caregivers from an initial cohort of 90. The sample size determination was based on a two-means power analysis, with the parameters set as follows: delta (μ1 – μ2) at 4.44, a standard deviation (SD) of 5.845, and a desired statistical power of 0.85. The estimated sample sizes were derived for a two-sample means test with a significance level (*α*) of 0.05. The hypothesis tested was Ho: m2 = m1, against the alternative hypothesis Ha: m2 ≠ m1. These calculations recommended an estimated total sample size (*N*) of approximately 64.22 (rounded off to 65) and an estimated sample size per group of approximately 32.11 to ensure the detection of statistically significant effects of the intervention. Participants were assigned sequential unique identifiers for unbiased allocation to the intervention or control group using Stata version 17 for random number generation. They were then distributed across the two groups to mitigate potential confounding factors and to maintain study integrity and validity. Recruitment, consent, and data collection were conducted in both English and Luganda to ensure clear communication and comprehensive understanding among participants, emphasizing the objectives of the pilot study in assessing recruitment feasibility, intervention adherence, and data collection efficiency. Figure 1 shows the participants’ flow chart.

Setting and location where data were collected: The study was conducted in village and sub-counties within the Wakiso district of Uganda. Caregivers were recruited and assigned to different groups. Participants in the intervention group were further subdivided into three groups. These groups met weekly at the local council chairpersons’ homes in the villages of Busukuma, Nansana, and Kayebe within the Wakiso district. Trial registration: Pan African Clinical Trials Registry (PACTR202211700581839).

### 2.1. Interventions—adaptation process of WHO-iSUPPORT

**Figure 2 •:**
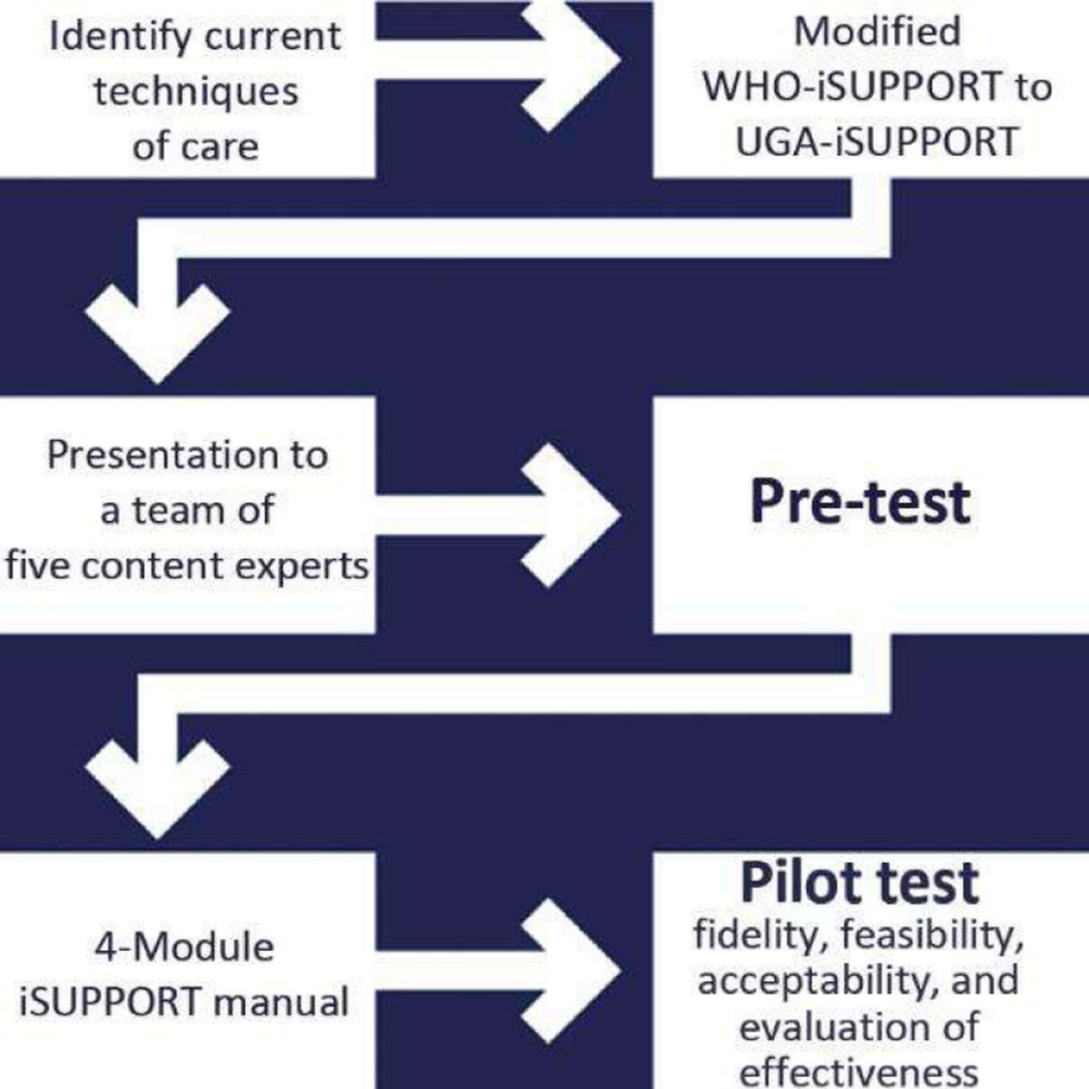
Adaptation of the WHO-iSUPPORT to UGA-iSUPPORT.

This study was structured around the Medical Research Council (MRC) framework for complex interventions as outlined by Craig et al. [19]. This framework was chosen because of its comprehensive and systematic approach to designing, implementing, and evaluating complex interventions in medical research. This involved a detailed development process, feasibility testing, piloting, and evaluation [20]. Each phase of the intervention was meticulously aligned with the MRC guidelines to ensure that the study design was conducive to effectively address the research questions and producing valid and reliable results (Figure 2).

First, the caregivers were asked to identify current techniques of care, suggest the most bothersome patient symptoms, outline components to be included in a training manual, and suggest the most feasible time, duration, and frequency of the intervention. From the caregiver responses, the original iSUPPORT manual was reduced to four modules, which were then translated into Luganda, back-translated, and presented to a team of five content experts, including a clinical psychologist, psychiatrist, public health expert, linguist, and a physician, for consensus. Figure 2 shows the work-flow of the adaptation process.

### 2.2. UGA-iSUPPORT pre-test

The pre-test phase aimed to evaluate various aspects of the UGA-iSUPPORT intervention from the caregiver’s perspective. The pre-test assessed caregiver comprehension of the UGA-iSUPPORT language, the applicability of the suggested skills to their day-to-day caregiving, usability of UGA-iSUPPORT, credibility of materials, caregiver involvement, relevance, and potential to motivate caregivers. Expert seminars were conducted with ten caregivers, three Village Health Team (VHT) members, and two local leaders in charge of older persons to gain a comprehensive understanding of these facets. The seminars continued until saturation was achieved, ensuring exhaustive and reflective feedback.

### 2.3. Intervention description

#### 2.3.1. Intervention group

In the intervention group, participants were divided into three subgroups of eleven. They attended four 90-minute weekly sessions of the UGA-iSUPPORT program. The Principal Investigator (PI) led these sessions, which comprised a blend of lectures, discussions, and hands-on activities. The intervention began with a foundational overview of dementia, followed by a module on the intricacies of caregiving, including effective communication and decision-making. The subsequent module highlighted self-care practices for caregivers, and the final module addressed daily care strategies, tackling common behavioral challenges associated with dementia, such as wandering away from home, hallucinations, refusal to eat and take medication, agitation, and irritability. The program’s design was multifaceted, with the aim of educating and actively involving caregivers in the practical aspects of their roles, such as meal presentation, providing frequent small meals, and establishing toileting routines to manage incontinence.

#### 2.3.2. Control group

In the control group, participants followed a modified standard-of-care protocol. This involved weekly five-minute phone calls over a period of four weeks, conducted by the PI. During these sessions, a structured protocol was used to enquire about patients’ health and wellness, while also providing empathetic support. Key areas included symptom variations, medication adherence, and overall health status. Additionally, if patients were taking medications for other illnesses, we enquired about their well-being and adherence to the prescribed treatments. This approach was informed by caregivers’ care preferences during the adaptation phase of the study.

### 2.4. Primary and secondary outcome measures

#### 2.4.1. Primary outcome measure

The study’s main goal was to assess the UGA-iSUPPORT program’s capacity to reduce caregiver distress. We employed the Kessler Psychological Distress Scale (K-10) for this quantitative evaluation, which demonstrated good reliability with a Cronbach’s alpha of 0.78. Assessments were systematically carried out at the intervention’s start and end, enabling a longitudinal study of the caregivers’ distress levels. The analysis accounted for initial differences among participants, offering insights into the temporal effectiveness of the program over four weeks. Notably, a K-10 score exceeding 21 was indicative of significant psychological distress.

#### 2.4.2. Secondary outcome measures

Beyond assessing caregiver distress, this study comprehensively examined secondary outcomes to understand the broader effects of UGA-iSUPPORT on caregivers. Central to this was evaluating caregivers’ life (QoL), utilizing the C-DMEQOL (*α* = 0.87) instrument—a measure specifically crafted for dementia caregivers. This assessment provides a detailed perspective on the impact of caregiving on overall well-being and life satisfaction, with higher C-DMEQOL scores correlating with improved QoL.

Additionally, the study investigated the intensity of depressive symptoms among caregivers using the Centre for Epidemiological Studies Depression Scale (CES-D [*α* = 0.79]), a tool renowned for its efficacy in detecting depression in epidemiological research. Recognizing that caregiving, especially in dementia contexts, is associated with increased psychological distress, depression was included as a critical secondary outcome in this study. A CES-D score of 16 or above indicates potential depressive symptoms.

### 2.5. Measures of feasibility, fidelity, and acceptability

### 2.5.1. Feasibility of UGA-iSUPPORT

To accurately assess the feasibility of the intervention, we implemented specific metrics such as recruitment consistency, facilitator performance, engagement, and interaction for evaluation. We defined “retention rate” as the percentage of participants who remained engaged from the start to the conclusion of the study, with a target of at least 80% retention to consider the intervention feasible. “Adherence rate” was measured as the percentage of participants who attended all scheduled sessions and completed the required activities, with an adherence rate of 75% or higher deemed satisfactory. For recruitment, the “time required to achieve the target sample size” was set with the expectation of enrolling all participants within an eight-week window. In addition, we monitored the number of participants who accessed and successfully completed all four modules of the intervention, aiming for a completion rate of 90% or higher. Comprehensive data collection procedures were established at baseline and at end line to ensure a robust assessment of these metrics

#### 2.5.2. Fidelity of UGA-iSUPPORT

To evaluate the fidelity of our intervention, we employed a dual approach involving specific fidelity checklists and direct observation [21].

##### Fidelity checklists:

We developed a checklist that details each critical component of the intervention. These checklists included items such as session duration, content covered, materials used, and interaction techniques employed by facilitators, as well as technical challenges like lack of visual aids, failure to get sitting space, etc.

After each intervention session, the PI completed these checklists, marking each element as addressed. This process served to self-assess and ensure that all aspects of the intervention were consistently and thoroughly delivered.

Additionally, an independent reviewer with a master’s degree in clinical psychology chose six random sessions for further evaluation using the same checklist to provide an objective measure of fidelity.

##### Direct observations:

Alongside the checklist, a subset of four intervention sessions was selected for direct observation by research team members who were not involved in the delivery of the intervention.

The observers were trained using a standardized protocol to ensure consistent and unbiased evaluation. They used a structured observation guide aligned with a fidelity checklist to note adherence to the intervention protocol and the quality of its delivery.

Observations also included notes on the participants’ engagement and interaction, providing qualitative insights into the delivery process.

#### 2.5.3. Acceptability of the UGA-iSUPPORT

The acceptability of the intervention was assessed qualitatively. Qualitative data were collected to investigate (i) whether caregivers found the language used was clear and were able to communicate the intended message, (ii) how useful and applicable the techniques were to daily life, and (iii) feedback about the mode of delivery.

##### Data analysis:

Data from the fidelity checklists and observation notes were analyzed to measure adherence to the intervention protocol. This process included calculating the percentage of completed checklist items per session and reviewing observer notes for deviations or notable events. The analysis provided a comprehensive view of the intervention’s execution, identifying areas of strong adherence and those requiring modifications or additional training for facilitators. The data analysis adhered to the intention-to-treat principle. To address any potential recruitment bias, baseline differences between the groups were carefully adjusted for in the subsequent analyses. Specifically, changes in scores from baseline to end line were analyzed using paired *t*-tests to compare within-group differences and independent *t*-tests to assess between-group differences. Furthermore, to account for any pre-existing variations between groups, a variance analysis (ANOVA) was conducted using baseline scores. Statistical tests were conducted at a 5% level of significance. Qualitative data were analyzed thematically.

##### Ethical considerations:

Ethical clearance was obtained from the Makerere University School of Medicine Research Ethics Committee (SOMREC, approved September 23, 2022) and the Uganda National Council for Science and Technology (UNCST, approved July 14, 2023) HS2909ES. The RCT was registered with the Pan African Clinical Trials Registry (PACTR2022117 00581839, approved November 24, 2022). We ensured that all participants were informed of the possibility of observation and consented to participate in the study. Confidentiality and anonymity were upheld in all fidelity assessments to safeguard participant privacy. During the course of the UGA-iSUPPORT sessions, some participants expressed their challenges of dealing with difficult patient situations by crying or expressing emotional upset. These occurrences were addressed through the communal sharing of personal experiences and the provision of encouragement by fellow group participants. Additionally, referrals to the VHT were made to provide extended support.

## 3. Results

### Overall description:

The study enrolled 65 caregivers of patients with ADRD and randomly assigned them to a 1:1 ratio between the intervention and control groups. Participant engagement remained stable throughout the study, with high scores for facilitator performance and learning environment, indicating effective program delivery. Notably, the intervention arm showed significant improvement.

### Psychological distress:

The mean K10 score decreased from 29.2 to 23.7 (*p* = 0.001). Depression severity: The mean CES-D score decreased from 33.4 to 25.6 (*p* = 0.001). QoL: The mean C-DEMQOL score increased from 81.1 to 89.4 (*p* = 0.001). These findings highlight the positive impact of the intervention on key outcomes compared with the control arm. Further details and implications are discussed in subsequent sections.

### 3.1. Background characteristics of participants

Participants were divided into two groups: a control group with 32 individuals and an intervention group with 33. The mean age of participants was comparable between the control and intervention groups, with means of 32 and 33 years, respectively. This difference was not statistically significant (*p* = 0.25). Conversely, the distribution of sex across groups revealed a significant disparity; the intervention group consisted predominantly of female participants (87.9%) compared to the control group, which had an equal distribution of males and females (50% each), resulting in a significant difference (*p* = 0.001). Marital status was similarly distributed among the groups, with the majority being single. Educational attainment differed, with a higher percentage of participants in the intervention group who had completed secondary education (66.7%) than in the control group (50%). The prevalence of health conditions was slightly lower in the intervention group, with 66.7% of participants reporting no health conditions, compared to 50% in the control group. The demographic details of the study are detailed in Table 1.

### 3.2. Impact of the UGA-iSUPPORT intervention on primary and secondary outcomes

#### 3.2.1. Psychological distress

At baseline, the intervention group reported a higher average K-10 score for psychological distress (29.2, SD = 1.1) than did the control group (25.8, SD = 1.3). The adjusted mean difference (AMD) was −3.4 (95% CI: −3.9 to −2.8, *p* = 0.036), indicating greater distress in the intervention group at the study’s outset.

A notable change was evident at the end of the study. The mean K-10 score for the intervention group decreased to 23.7 (SD = 0.7), while the control group’s mean score increased to 27.0 (SD = 0.9). This resulted in an AMD of 3.3 (95% CI: 2.9–3.7, *p* = 0.001), signifying a substantial reduction in psychological distress among intervention group members.

#### 3.2.2. Quality of life

Baseline average QoL scores were higher in the intervention group (81.1, SD = 0.9) than in the control group (78.9, SD = 1.3). The AMD was −2.1 (95% CI: −2.7 to −1.6, *p* = 0.065), suggesting a non-significant tendency toward lower baseline QoL in the intervention group.

At the end of the study period, a notable enhancement in QoL was observed in the intervention group. The average scores increased to 89.4 (SD = 1.8), while the control group’s scores decreased to 77.5 (SD = 2.3). The AMD was −11.9 (95% CI: −12.9 to −10.9, *p* = 0.001).

#### 3.2.3. Depression scale

Baseline-mean depression scores, assessed using the CES-D scale, were comparable across groups: 33.4 (SD = 1.2) for the intervention group and 34.9 (SD = 1.5) for the control group. The AMD was 1.5 (95% CI: 1.1–1.9, *p* = 0.321), indicating no significant difference initially.

However, at the end of the study period, a notable difference was observed. The intervention group’s average CES-D score decreased to 25.6 (SD = 2.3), while the control group’s score slightly dropped to 33.7 (SD = 3.1). The AMD was 8.1 (95% Cl: 7.6–8.6, *p* = 0.001), signifying a significant decrease in depression severity in the intervention group. More details of the study are provided in Table 2.

### 3.3. Findings on feasibility, fidelity, and acceptability of the UGA-iSUPPORT and control group

#### 3.3.1. Intervention feasibility

Intervention feasibility was assessed in five dimensions: Recruitment consistency, facilitator performance improvement, engagement and interaction, environment and logistics, and method of delivery. A more elaborate distribution of the feasibility of UGA-iSUPPORT is presented in Tables 3 and 4.

Regarding recruitment consistency, the intervention successfully maintained consistent attendance, with each session having at least ten participants (ranging from 10 to 11), demonstrating the effectiveness of our recruitment and retention strategies. This steady attendance, as shown in Table 1, emphasizes the program’s capacity to attract and keep participants engaged. Remarkably, the attrition rate was exceptionally low, with only one of the original 33 participants (3.03%) not completing the program, which further supports the success of our engagement strategies.

Regarding facilitator performance improvement, the facilitator’s performance showed progressive improvement, with ratings in communication, engagement, and knowledge of the subject matter consistently increasing. In the final session, perfect scores were obtained for several areas.

Participant engagement evolved from “Medium” to “High”, during the course of the study, suggesting that the intervention became more engaging or relevant to the participants over time. Active participation in activities also scored consistently well (4/5–5/5).

Regarding environment and logistics, high scores for environmental factors (5/5 for a conducive learning environment) and the absence of technical issues throughout the sessions reflected a well-organized and conducive setting for the intervention.

Findings of the method of delivery show that content delivery was rated highly, with clear, audible presentations, and organized delivery. The information provided was consistently accurate and up-to-date, and the content was tailored to the audience’s needs.

The intervention was feasible in terms of consistent participant engagement, effective facilitation, conducive environment, and well-structured content delivery (Table 3).

##### UGA-iSUPPORT intervention fidelity:

Intervention fidelity was assessed in five domains that included session conduct, facilitator performance, participant engagement, technical challenges, and methods of delivery.

Regarding session conduct, all sessions were started and concluded on time, indicating excellent adherence to the schedule. All the planned topics were covered, and interactive elements were included in each session.

The facilitator’s performance improved over the sessions, with scores on communication, engagement, knowledge, and interaction progressively increasing, culminating in perfect scores in the final session.

Participant engagement was rated as “Medium” initially and improved to “High” in the later sessions. The questions were consistently addressed satisfactorily, with scores of 4/5 improving to 5/5 in later sessions.

Regarding technical challenges, no technical issues were encountered during these sessions.

General observations and methods of delivery: The independent rater (IR) mentioned

“The environment was consistently conducive to learning. Facilitator-participant interaction was positive and respectful, with improvements in the nonverbal cue response.’’ and “Content delivery was clear, organized, and tailored to the audience’s needs.”

The intervention was observed to be feasible in terms of consistent participant engagement, effective facilitation, conducive environment, and well-structured content delivery. This indicates a strong potential for the successful implementation of the program.

### 3.4. Control group fidelity

Regarding protocol adherence, out of four scheduled phone calls per individual, approximately 96% commenced on time, demonstrating robust adherence to the protocol. The remainder began either five minutes late or were rescheduled by the participants for a later time. Nearly all scheduled calls took place, with the exception of two that did not occur because of the deregistration of the associated phone numbers.

On duration consistency, approximately 90% of the calls adhered to the planned five-minute duration. Variations in call duration, such as three minutes, six minutes, or phone being off, indicate some deviations from the protocol. Calls that did not reach four minutes were rescheduled by the PI for another time.

### 3.5. Control group feasibility

On participant responsiveness, a small number of late starts, abbreviated sessions, and rescheduling (4.0%) occurred in the control group; however, these instances were not substantial enough to pose challenges to participant engagement.

On rescheduling and non-participation, the deregistration of some participants’ phone numbers (2) and two instances of rescheduling indicate challenges in ensuring participant availability. However, these issues likely did not affect the feasibility of the control arm’s modified standard of care.

Overall, there was a high level of adherence to the protocol regarding start times, and most calls met the duration criteria.

### 3.6. Acceptability of the UGA-iSUPPORT intervention

This was done qualitatively, where participants in the intervention arm were asked to give feedback about UGA-iSUPPORT, and is further represented in Table 4. Five themes were identified: language clarity, literacy considerations, health worker distribution, recognition of care needs, and the caregiver-centric approach.

The majority of the participants (80%) found the language used in the study to be clear and straightforward. Nonetheless, a few participants suggested the need for greater simplification, especially when explaining intricate concepts, such as alternative feeding methods, activities of daily living, and self-care strategies for caregivers. For instance, one participant noted

“The word feeding alternatives is difficult to understand, we need to break it down to options to poor feeding.” (CG female)

Another participant suggested,

“When you are talking about activities of daily living, what do you mean? The way it is we may not understand it, add examples in the explanation so that we know what you are talking about.” (CG male)

All participants recognized the intervention’s value but pointed out its potential limitations among illiterate individuals, particularly if the intervention was self-administered rather than delivered by a caregiver or a VHT. This implies that the efficacy of the intervention may be limited to populations with lower literacy levels. For instance, one caregiver noted

“The program is good and easy to understand if someone with experience is to deliver it. However, if we are to read for ourselves, it will only be possible by those who have at least learned how to read and write and that will live out those with no education.”(CG male)

The suggestion to distribute intervention materials through VHTs indicates a preference for more widespread and community-based dissemination methods.

“We are familiar with the VHTs, they know us and sometimes even give some medicines like de-wormers, malaria drugs to us and our patients. It will be better if they are the ones to offer the intervention next time. We know them and they know us of course they need to be supervised by you people (the facilitators)” (CG female)

Participants expressed appreciation for the intervention’s focus on caregivers, indicating that it successfully addressed previously unmet needs. For example, one caregiver mentioned

“The program has been very good. I used to think I am not allowed to even have a break and it would make me feel bad and sometimes hate my patient, but now I even learned how to create sometime for myself to relax.” (CG female). This sentiment reflects the potential of interventions to enhance caregiver support and well-being.

The positive feedback on the intervention’s focus on caregivers, rather than just patients, suggests that the intervention successfully resonates with its intended audience and fills a critical gap in caregiver support. A VHT mentioned

“This is a very good program; we have not seen any program for caregivers and yet they too suffer a lot. We have observed how some of them struggle to care for these patients. This is really good.” (VHT female)

Overall, the acceptability data suggest that while the UGA-iSUPPORT intervention is well received and fills a crucial gap, enhancements in language simplicity and accessibility, particularly for non-literate caregivers, could increase its effectiveness and reach.

## 4. Discussion

This study aims to adapt and evaluate the WHO-iSUPPORT program on feasibility, fidelity, acceptability, and impact on psychological distress; QoL; and depression severity for caregivers of people with dementia in Uganda. Addressing this critical gap, this study contributes to the sparse evidence on interventions for dementia caregivers in LMICs, where the burden of dementia is notably rising [1,3,4]. The findings are promising, demonstrating that UGA-iSUPPORT is both feasible and effective, showing significant improvements in caregivers’ psychological well-being, the severity of depression, and QoL. Moreover, the study established UGA-iSUPPORTs high fidelity and feasibility, underscoring its viability as a culturally sensitive and scalable intervention to bolster the mental health and well-being of dementia caregivers in LMICs. This discussion delves into the implications of these results for dementia care practices in Uganda and similar contexts while also considering the study’s limitations and proposing directions for future research.

### 4.1 Key findings and interpretations

#### 4.1.1 Reduction in caregiver psychological distress and depression severity

The significant decrease in psychological distress and depression among the intervention group underscores the effectiveness of the UGA-iSUPPORT program in addressing mental health challenges associated with dementia caregiving. The UGA-iSUPPORT model, which provides comprehensive and culturally sensitive support, meets caregivers’ complex emotional and informational needs. This method aligns with the increasing global research that stresses the need for specialized mental health interventions for caregivers, which not only improves their well-being but also the quality of care for dementia patients [9].

Comparative studies in developed nations have reported similar benefits from caregiver support programs, suggesting that dementia caregivers face universally challenging circumstances, although these vary in complexity based on socioeconomic and cultural contexts [10]. For example, high-income areas often utilize digital technologies and established healthcare systems to aid caregivers, an approach that is less viable in many LMICs because of technological and infrastructural limitations [11]. Nonetheless, the achievements of UGA-iSUPPORT echo those of other LMICs, where culturally tailored community-based interventions have likewise shown significant mental health improvements for caregivers [12,15]. These findings confirm the global necessity for caregiver support and the critical role of cultural and contextual considerations in the development and implementation of such programs.

#### 4.1.2. Enhanced QoL among caregivers

The observed enhancement in QoL among caregivers participating in the UGA-iSUPPORT program’s intervention group highlights its extensive benefits. These benefits are vital in the face of the global dementia care challenge, where caregivers frequently endure significant emotional and physical strain. The active engagement and skill building offered by UGA-iSUPPORT seem to be key in fostering a supportive community for caregivers, which is essential for their overall well-being. This focus on social support and community engagement is reflected in research from both developed nations and LMICs, underscoring the universal importance of such programs in dementia caregiving scenarios and landscapes [12,15].

These findings indicate that the UGA-iSUPPORT intervention effectively addressed the unmet needs of caregivers and enhanced their coping skills and well-being. Our findings are consistent with those of previous studies demonstrating the positive effects of comprehensive support programs for caregivers of people with dementia in different settings [9, 22]. However, unlike these studies, our intervention was physically delivered in groups, which increased accessibility and scalability in resource-limited settings.

Feasibility and fidelity of the UGA-iSUPPORT: Notably, our results showed that the intervention was feasible, as evidenced by the high retention rate, stability of participant engagement, and improvement in facilitator performance across the sessions. The intervention was delivered with high fidelity, as indicated by the adherence to the protocol, quality of the content, and satisfaction of the participants. These findings suggest that the UGA-iSUPPORT intervention was acceptable and appropriate for the Ugandan context, where resources are limited, and the delivery of health interventions faces numerous logistical challenges. Our findings are consistent with previous studies that have reported the feasibility and fidelity of online or blended interventions for caregivers of people with dementia in different settings [23,24].

However, unlike these studies, which borrowed from cognitive behavioral therapy and family theory, our intervention was adapted from the iSUPPORT program developed by the WHO, which is a global resource for dementia care [8]. The high scores for engagement, interaction, and a conducive learning environment further illustrate the program’s acceptability among participants, highlighting the importance of cultural and contextual adaptation in the intervention design [25]. Our study further contributes to the literature on the adaptation and implementation of the iSUPPORT program, which was developed by WHO as a global resource for dementia care [8].

#### 4.1.3 Contradictions in control group outcomes

The decline in psychological well-being observed in the control group of this study reveals a paradoxical but essential insight into minimal interventions for dementia caregivers. This suggests that increasing caregivers’ awareness of their challenges without offering effective coping strategies may heighten their stress unintentionally. This finding is consistent with other research warnings regarding the potential adverse effects of well-intentioned but inadequate interventions [26]. This underscores the need for a careful balance in developing caregiver support programs, ensuring that information is coupled with tangible support to avoid adding stress to caregivers [27].

This paradoxical effect echoes the “forewarning” concept in the psychological literature, where awareness does not automatically lead to adaptive behavior change, particularly in the absence of solid coping strategies [28]. In caregiver support, this indicates that interventions should be comprehensive, providing not only awareness but also practical tools and strategies for effective caregiving. The importance of a nuanced approach in designing interventions is further emphasized by research that reveals the intricate dynamics of caregiving stress and its effects on mental health [29]. Such research supports the development of interventions that are informative and empowering, equipping caregivers with the necessary resources and skills to fulfill their roles with resilience and effectiveness like the UGA-iSUPPORT.

#### 4.1.4. Limitations

This study presents certain limitations that warrant caution in the interpretation of results. First, the small sample size and brief follow-up period might restrict the statistical significance and durability of the effects of the intervention. Second, the recruitment of participants from a single peri-urban locale could hinder the applicability of the findings across different areas or contexts. Subsequent research should aim to mitigate these issues by incorporating broader and more varied samples as well as extending the duration of follow-up.

## 5. Conclusions

This study tackles the urgent need for caregiver support in LMICs and introduces UGA-iSUPPORT as a viable, potent, and culturally attuned intervention. The principal outcomes revealed substantial decreases in caregivers’ psychological distress and depression levels, along with enhancements in QoL. These findings underscore the program’s capacity to fulfill the intricate emotional and informational requisites of caregivers, emphasizing the need for specialized, context-specific mental health interventions. The program’s practicability and authenticity in the Ugandan context, as evidenced by high retention and satisfaction rates, further affirms its potential for wider application in comparable settings with limited resources. Nevertheless, the study also detected an unexpected impact of minimal intervention on the control group, suggesting that heightened awareness of caregiving challenges without adequate coping mechanisms could inadvertently amplify stress. Despite limitations such as a modest cohort size and brief follow-up period, this pilot RCT lays the groundwork for the efficacy of UGA-iSUPPORT, promoting further investigation into its adaptability and long-term viability in various environments.

### 5.1. Policy implications

The findings advocate the integration of caregiver support programs into national health systems, emphasizing the need for policies that recognize and address mental health challenges faced by caregivers of individuals with dementia. Investing in caregiver support not only improves the QoL of caregivers but also has broader societal benefits, including the sustainability of care systems and the overall health and well-being of communities. Policymakers should prioritize the development and scaling of interventions such as UGA-iSUPPORT, ensuring that they are accessible, culturally relevant, and adequately funded.

### 5.2. Future directions

Research: Future studies should look at the effectiveness and the cost-benefit analysis of undertaking such interventions in preparation for scaling up in low-resource settings like Uganda. Furthermore, longitudinal studies are needed to evaluate the enduring effects of such caregiver interventions as well as comparative studies to assess various modes of delivery.

### 5.3. Implementation

The results support the nationwide implementation of programs similar to UGA-iSUPPORT, highlighting the necessity of educating healthcare professionals and community health workers on administering these interventions within the current health systems.

## Figures and Tables

**Table 1 •:** Baseline characteristics

Factor	Control arm	Intervention arm	*p* Value
Age: Mean (SD)	32 (49.2)	33 (50.8)	
	36.7 (16.0)	32.5 (12.7)	0.25

Sex^a^			
Female	16 (50)	29 (87.9)	0.001
Male	16 (50)	4(12.1)	

Marital status			
Divorced	1 (3.1)	1 (3.0)	0.937
Married	13 (40.6)	12 (36.4)	
Single	18 (56.2)	20 (60.6)	

Level of education			
Primary	10 (31.2)	8 (24.2)	0.340
Secondary	16 (50)	22 (66.7)	
Tertiary	6 (18.8)	3 (9.1)	

Children			
No	8(25)	5(15.2)	0.665
Yes	24 (75)	28 (84.8)	

Health condition			
No	16 (50)	22 (66.7)	0.28
Yes	16 (50)	11 (33.3)	

a Significantly different at baseline.

**Table 2 •:** Impact of the UGA-iSUPPORT intervention on primaiy and secondary outcomes

Outcome	Intervention arm	Control arm	AMD	95% Cl	*p* Value
	Mean (SD)	Mean (SD)			
Depression scale					
Baseline CES-D scores	33.4 (1.2)	34.9 (1.5)	1.5	1.1; 1.9	0.321
End line CES-D scores	25.6 (2.3)	33.7(3.1)	8.1	7.6; 8.4	0.001
Quality of life					
Baseline C-DEMQOL scores	81.1 (0.9)	78.9 (1.3)	−2.1	−2.7; −1.6	0.065
End line C-DEMQOL scores	89.4 (1.8)	77.5 (2.3)	−11.9	−12.9; −10.9	0.001
Psychological distress					
Baseline K-10 scores	29.2 (1.1)	25.8 (1.3)	−3.4	−3.9; −2.8	0.036
End line K-10 scores	23.7 (0.7)	27.0 (0.9)	3.3	2.9; 3.7	0.001

**Table 3 •:** Feasibility of the UGA-iSUPPORT intervention

Feasibility aspect	Details	Assessment
Recruitment consistency	Number of participants remained stable (32–33) across sessions.	Highly feasible
Facilitator performance	Progressive improvement in communication, engagement, and subject knowledge. Perfect scores in the final session.	Highly feasible
Engagement and interaction	Evolved from “Medium” to “High”. Active participation scores ranged from 4/5 to 5/5.	Highly feasible
Environment and logistics	High scores (5/5) for conducive learning environment. No technical issues were reported.	Highly feasible
Method of delivery	Content delivery was dear, audible, and well organized. Information was accurate and up-to-date.	Highly feasible

**Table 4 •:** Acceptability of the UGA-iSUPPORT

Acceptability theme	Feedback summary	Suggestions for improvement
Language clarity	80% found the language clear but suggested simplification for terms like “feeding alternatives, activities of daily living, adopt, and connected”.	“Break down complex terms, add examples”.
Literacy considerations	Acknowledged usefulness but highlighted limitation to literate individuals.	“Consider non-literate individuals in the delivery approach”.
Distribution via health workers	Preferred community-based dissemination through Village Health Teams (VHTs).	“Utilize VHTs for more effective distribution”.
Recognition of caregivers’ needs	High appreciation for focusing on caregivers, addressing unmet needs like self-care.	“Continue to emphasize caregiver support”.
Caregiver-centric approach	Positive feedback for the program’s focus on caregivers, filling a critical support gap.	“Maintain and enhance the caregiver-centric approach”.

## Data Availability

Data supporting these findings are available within the article, at https://doi.org/10.20935/MHealthWellB7292, or upon request.
